# The effects of green cardamom on blood glucose indices, lipids, inflammatory factors, paraxonase-1, sirtuin-1, and irisin in patients with nonalcoholic fatty liver disease and obesity: study protocol for a randomized controlled trial

**DOI:** 10.1186/s13063-017-1979-3

**Published:** 2017-06-07

**Authors:** Milad Daneshi-Maskooni, Seyed Ali Keshavarz, Siavash Mansouri, Mostafa Qorbani, Seyed Moayed Alavian, Mahtab Badri-Fariman, Seyed Ali Jazayeri-Tehrani, Gity Sotoudeh

**Affiliations:** 1 Department of Community Nutrition, School of Nutritional Sciences and Dietetics, Tehran University of Medical Sciences, Tehran, Iran; 2 Department of Clinical Nutrition, School of Nutritional Sciences and Dietetics, Tehran University of Medical Sciences, Tehran, Iran; 3 National Iranian Oil Company (NIOC) Central Hospital, Tehran, Iran; 4 Non-Communicable Diseases Research Center, Alborz University of Medical Sciences, Karaj, Iran; 5 Baqiyatallah Research Center for Gastroenterology and Liver Diseases (BRCGL), Baqiyatallah University of Medical Sciences, Tehran, Iran

**Keywords:** Trial, Protocol, Nonalcoholic fatty liver disease, Green cardamom, Overweight, Obesity

## Abstract

**Background:**

The relationship between dietary components and nonalcoholic fatty liver disease (NAFLD) needs to be further investigated. The potential health benefits of cardamom have been found in some studies. Cardamom showed beneficial effect on hepatomegaly, dyslipidemia, and fasting hyperglycemia in animals. However, some adverse effects of cardamom have been reported in animals. No previous human study had been conducted on the effects of cardamom in NAFLD. This study aims to determine the effects of green cardamom (*Elettaria cardamomum*) supplementation on blood glucose indices, lipids, inflammatory profiles, and liver function, especially by examining irisin, paraxonase-1 (PON1) and sirtuin-1 (Sirt1) in obese patients with NAFLD.

**Methods:**

This trial is to be conducted at the polyclinic of the National Iranian Oil Company (NIOC) Central Hospital, Tehran. Eighty obese patients with NAFLD will be selected according to the eligibility criteria. The NAFLD diagnosis method is ultrasonography. Patients will be randomly divided into two groups by a random-number table (cardamom and placebo groups, two 500-mg capsules, three times/day, taken with meals for 3 months, follow-up monthly). General characteristics, dietary intakes (at the beginning, middle, and end), and physical activity (at the beginning and end) will be assessed using a general, 24-h food recall, and short-form International Physical Activity Questionnaires (IPAQ), respectively. Lifestyle advice will be presented to both groups identically. At the beginning and the end, anthropometrics (weight, height, and waist circumference), blood pressure, extent of fatty liver, and blood biomarkers, including serum glucose indices (fasting blood sugar (FBS)) and insulin (FBI), homeostasis model assessment-insulin resistance (HOMA-IR), Quantitative Insulin Sensitivity Check Index (QUICKI)), lipids (triglyceride (TG), low-density lipoprotein-cholesterol (LDL-c), high-density lipoprotein-cholesterol (HDL-c), total cholesterol (TC)), inflammatory markers (highly sensitive C-reactive protein (hs-CRP), tumor necrosis factor-alpha (TNF-α), interleukin-6 (IL-6)), liver enzymes (alanine transaminase (ALT), aspartate transaminase (AST)), irisin, PON1, and Sirt1, will be determined.

**Discussion:**

This trial would be the first to assess the effects of green cardamom on several blood factors, including glucose indices, lipids, inflammatory markers, liver enzymes, irisin, PON1, and Sirt1, and blood pressure and anthropometry in obese patients with NAFLD. Further study of cardamom’s potential in improving NAFLD is suggested.

**Trial registration:**

Iranian Registry of Clinical Trials (IRCT), ID number: IRCT2015121317254N4. Registered on 27 December 2015.

**Electronic supplementary material:**

The online version of this article (doi:10.1186/s13063-017-1979-3) contains supplementary material, which is available to authorized users.

## Background

NAFLD is engendered due to the accumulation of large amounts of triglycerides in liver cells (5% <) [1]. The prevalence of NAFLD is currently increasing [2] (an average of 30% in Iranian adults [3, 4]). NAFLD prevalence in obese (Body Mass Index (BMI) ≥25) and nonobese (BMI <25) adults has been reported as being approximately 65–85% and 15–20%, respectively [5]. The pathology of NAFLD is a two-stage phenomenon, including the accumulation of fat in hepatocytes and subsequent hepatic steatosis in the first stage, and nonalcoholic steatohepatitis (NASH) in the second stage. Insulin resistance plays a key role in both stages and oxidative stress and proinflammatory cytokines are major stimulating components of the pathology [6]. NAFLD, being a major health problem, includes a wide range of clinical symptoms (from the asymptomatic fatty liver to severe inflammation along with fibrosis and sometimes cirrhosis). Often, these patients are insulin-resistant [7]. Insulin resistance is directly correlated with the content of fat in the liver. Nuclear factor kappa-light-chain-enhancer of activated B-cells (NF-κB) is a protein complex responsible for cytokine production and cell survival. Known triggers of NF-κB include reactive oxygen species (ROS), tumor necrosis factor-alpha (TNF-α), interleukin-1 beta (IL-1β), bacterial lipopolysaccharide, isoproterenol, cocaine, and ionizing radiation. NF-κB activation upregulates the production of proinflammatory cytokines that affect both local and systemic insulin action. This means that inflammation, adipokines, oxidative stress, and lipid metabolites can affect insulin sensitivity, but are not necessarily directly associated with intrahepatic fat content [5]. Most patients with NAFLD have metabolic syndrome characteristics, including being overweight and obese, impaired glucose tolerance, hyperlipidemia and hypertension [8, 9]. Other risk factors of NAFLD are age, family history, malnutrition, severe weight loss, the consumption of certain medicines, certain diseases [10], and gastrointestinal microbiota [11].

Along with increasing oxidative stress, both the activity and production of bonded-to-high-density lipoprotein-cholesterol (HDL-c) blood PON1 is suppressed in patients with NAFLD [12, 13]. PON1 has an anti-inflammatory role by interfering with the production of inflammatory factors [12]. PON1 is activated by peroxisome proliferator-activated receptor gamma (PPAR-γ) [14] and, according to previous studies, the intake of dietary polyphenols (particularly flavons) is associated with increasing PPAR-γ [15–17].

Irisin is a newly identified myokine related to exercise. Recently, it was reported that serum irisin levels have shown associations with intrahepatic triglyceride content which is indicative of it playing an important hepatic role [18]. According to recent studies, irisin plays a role in insulin sensitivity [18–22] through effects on glucose and lipid metabolism [19, 23] and increased thermogenesis [19, 20, 24]. Its secretion can be increased by lifestyle modifications, regular exercise [20–22, 24, 25], and stress [21]. In a human study, serum irisin levels were inversely associated with the triglyceride (TG) content in the liver and liver enzymes in obese adults (aged 40 years and older) with NAFLD [26].

Sirtuins have seven categories, of which Sirt1 has mainly been identified and studied in humans [27]. Sirt1, as a histone deacetylase class III, decreases oxidative stress indirectly through catalase and superoxide dismutase (SOD) [28]. Sirt1 activation has beneficial health consequences such as improved insulin sensitivity, obesity reduction, increased mitochondrial function, decreased glucose levels, and increased physiological functions [29, 30]. Sirt1 plays an important role by increasing PPAR-γ co-activator-1 alpha (PGC-1α) [27, 29]. Usually, polyphenols activate Sirt1 and, subsequently, PGC-1α also inhibits NF-κB [29, 31].

Dietary polyphenols including derivatives and isomers of flavones, isoflavones, flavonoids and catechins and phenolic acids have antioxidant and anti-inflammatory properties [32]. Green cardamom (*Elettaria cardamomum*) contains phenolic and flavonoid compounds, such as flavonols (quercetin and kaempferol), flavone (luteolin) and anthocyanidin (pelargonidin) [33], that are inhibitors of NF-κB [33–36]. Isoflavones and flavonoids, such as quercetin, resveratrol [37], and kaempferol, increase the activity of PGC-1α [38]. On the other hand, irisin secretion increases in response to increasing PGC-1α [25]. Owing to the effect of dietary polyphenols on PGC-1α, it is possible that green cardamom polyphenols will be able to influence thermogenesis and improve insulin resistance. Moreover, in cell studies, irisin effectively prevents hepatic steatosis by lipogenic gene expression changes and oxidative stress inhibition [18]. In addition, irisin leads to fibroblast growth factor-21 (FGF-21) upregulation through the PPAR signaling pathway, especially PPAR-α [22], which can subsequently improve insulin sensitivity and hepatic steatosis [22, 26].

Lifestyle changes—including gradual weight loss and increasing physical activity—are common ways to reduce and treat NAFLD [39–43]. Since achieving weight loss and maintaining a reduced weight for a long time is difficult [44], it seems that dietary changes may be a proposed therapeutic approach for these patients [45]. Some studies were conducted on green cardamom’s role in health as a spice. The effects of cardamom reported by those studies are blood pressure lowering, fibrinolysis enhancing, antispasmodic effects, and gastroprotective, antibacterial, antioxidant [46], anti-inflammatory, anti-food poisoning, carminative, diuretic, expectorant, heart-function improving, anticarcinogenic [47], and antiplatelet aggregation effects [48]. Cardamom volatile oil contains terpenes, esters, flavonoids and other compounds. The major constituents of the volatile oil of cardamom include about 36% 1,8-cineole, 31% alpha-terpinil acetate, 12% limonene, 3% sabinene and others. Most studies have been conducted on the 1,8-cineole compound. The reported effects are apoptotic, the inhibiting of cytokines, prostaglandins, leukotrienes, and nitric oxide (inhibition of cyclooxygenase-2 (COX-2) and inducible nitric oxide synthase (iNOS)), TNF-α and IL-1β inhibition, liver necrosis reduction, cardiovascular effects (blood vessel relaxation), as well as anticholinergic effects and the blocking of muscarinic receptors [49].

The hypothesis is that the antioxidant, anti-inflammatory, antibacterial, and hypolipidemic activities of cardamom can improve NAFLD. The effects of cardamom on Sirt1 secretion and, subsequently, PGC-1α, irisin, and insulin sensitivity are not clear. Moreover, its effects on PPAR-γ and, subsequently, serum PON1 levels need further study. With an awareness that NAFLD increases in overweight or obese populations, and the lack of human studies on the effects of green cardamom, this clinical trial study was planned to determine its impact on blood glucose indices, lipids and inflammatory profiles, liver function, and insulin resistance, especially through irisin, PON1, and Sirt1.

## Methods

### Study design and objectives

A double-blind randomized clinical trial design is to be used in this study.

### Objectives

- × Compare the mean serum lipid (triglycerides (TG), total cholesterol (TC), low-density lipoprotein (LDL), high-density lipoprotein (HDL)) and glucose indices (fasting blood sugar (FBS), fasting blood insulin (FBI), homeostasis model assessment-insulin resistance (HOMA-IR), Quantitative Insulin Sensitivity Check Index (QUICKI)) between the two groups and within each group, before and after intervention
- × Compare the mean serum inflammatory factors (TNF-α, interleukin-6 (IL-6), highly sensitive C-reactive protein (hs-CRP)) between the two groups and within each group, before and after intervention
- × Compare the mean of serum PON1, sirt1, and irisin between the two groups and within each group, before and after intervention

### Inclusion criteria

- × NAFLD diagnosed by a radiologist and a hepatologist using ultrasonography, and divided into one of three categories (mild, moderate or severe degree)
- × Age 30–60 years
- × Overweight or obese (25 ≤ BMI < 35)
- × Informed consent signed and dated by the subject and investigator

### Exclusion criteria

- × History of alcohol consumption during the preceding 12 months, based on patient’s own evidence
- × Suffering from cognitive impairment or other psychotic illnesses, as diagnosed by a psychiatrist
- × Severe depression, lacking the ability to cooperate and answer questions
- × Diagnosed pathological conditions affecting the liver such as viral hepatitis, acute or chronic liver failure, cholestasis, liver transplantation, habitual abuse of nonsteroidal anti-inflammatory drugs, antibiotics, antisecretory drugs causing achlorhydria within 3 months before the study, corticosteroids, amiodarone, valproate, prednisone, tamoxifen, perhexiline and methotrexate, rapid weight loss, diabetes, heart failure, thyroid disorders, kidney disease, respiratory failure, psychological disorders, hereditary hemochromatosis and Wilson’s disease, alpha-1 antitrypsin deficiency, autoimmune diseases, celiac disease, use of liver fat inducers, and hormonal drugs
- × Acute systemic disease, cystic fibrosis, muscular dystrophy, protein malnutrition, a history of gastrointestinal surgery, neurological disorders, structural abnormalities of the gastrointestinal tract
- × NAFLD caused by secondary factors including drugs, surgical procedures, environmental toxins, and total parenteral nutrition (TPN)
- × Conditions leading to physical inactivity (disability)
- × Uncontrolled hypertension (>140/90 mmHg)
- × Any diagnosed malignancy
- × Breast feeding, pregnant, and/or planning for pregnancy in the following 3 months
- × Professional athlete or doing regular exercise
- × Treatment with statins, antihypertensive drugs, ursodeoxycholic acid, probiotics, and multivitamin-mineral and antioxidant supplements during the past 3 months
- × Surgery for weight loss in the past year and weight loss in the past 3 months
- × Intake of drugs that interact with cardamom including aspirin, anticoagulants (warfarin, heparin) and antiplatelet (clopidogrel), nonsteroidal anti-inflammatory (ibuprofen or naproxen), blood pressure-lowering drugs, central nervous system (CNS) depressants (benzodiazepines such as lorazepam or diazepam, barbiturates such as phenobarbitals, narcotics such as codeine, some antidepressants and alcohol), anaesthetics, antibiotics, anticancer agents, anticholinergics, antifungals, cyproheptadine, diuretics (loop), estrogen, indomethacin, muscarinic agents, pain relievers and prednisolone
- × Taking a multivitamin-mineral or antioxidant supplement at least once a week during the study
- × Not taking more than 10% of prescription supplements

### Study subjects

Patients with NAFLD attending the polyclinic of the NIOC Central Hospital in Tehran are to be invited to the study. The patients will be diagnosed by a radiologist and a hepatologist as having fatty liver, using ultrasonography. Patients should meet the eligibility criteria. After their identification, the patients will be referred to the principal investigator.

First, the goals, methods, and benefits of the intervention will explained and Informed Consent Forms signed. The general questionnaire, short-form International Physical Activity Questionnaire (IPAQ) and 24-h food recall questionnaire are to be completed by interviews. Necessary education and guidelines concerning lifestyle modification, will then be given [45], including a weight-loss diet (5% weight loss during the study [50]) and increasing physical activity (aerobic with moderate intensity, at least 3 times per week, 30–45 min [51]).

Anthropometric measurements, including weight, height and waist circumference are to be assessed by using a digital scale, stadiometer, and nonelastic tape, respectively.

A 24-h food recall questionnaire is to be completed at the beginning, after 1.5 months, and at the end. Blood pressure (systolic and diastolic) will be measured at the beginning and the end with a manometer. Ten milliliters of blood at the beginning and 10 ml at the end are taken from a peripheral vein after a 12-h overnight fast. After centrifuging for 20 min (3000 *g*), the serum samples will be frozen simultaneously and stored at −80 °C until analyzed. At the end, the evaluated outcomes are to be presented to the patients privately.

### The sample size

The sample size was calculated using the “two mean comparison formula” and was related to a previous study [52] which had assessed the effects of cardamom on lipid profiles. In this study, the errors I and II are considered to be 0.05 and 0.2, respectively. The mean difference of TG between the groups was 5 mg/dl, and the standard deviation of each group was 8 mg/dl. The sample size was determined to be 40 subjects in each group. In total, 80 patients were invited and randomly divided into two groups as follows:

1. Forty overweight or obese patients with NAFLD will be given lifestyle change recommendations (weight-loss diet (5%) during the study and increasing physical activity) and a green cardamom supplement for 3 months
2. Forty overweight or obese patients with NAFLD will be given lifestyle change recommendations (weight loss diet (5%) during the study and increasing physical activity) and a placebo supplement for 3 months

## Randomization and intervention

Patients are divided into two equal groups using block randomization. Stratified randomization will be used to control age and gender distribution. In this study, the ratio of green cardamom and placebo supplementation groups is 1:1. The block randomization is performed by an assistant and the intervention allocation is blinded for both the investigator and subjects. The participants are randomly placed into two groups receiving whole green cardamom powder or toast powder supplements. Cardamom and placebo capsules are prepared by the Traditional Medicine Research Center (TMRC), Iran University of Medical Sciences. Each capsule contains 0.5 g of whole green cardamom or toast powder. Placebo and cardamom capsules are similar in shape, size, and color. At the beginning of the study, all placebo capsules are to be placed near the cardamom capsules so that the patients are able to smell them. Even though some cardamom volatile oils may be absorbed by the placebo capsules, this amount is considered negligible compared to the amounts that affect health indicators. The type of supplements is blinded as A and B packages for investigators and patients.

According to previous trials, the dose of whole green cardamom powder was determined to be 3 g per day [46, 52, 53]; two capsules were consumed with each meal.

The voucher number of green cardamom is *Elettaria cardamomum* (L.) Maton, Family: Zingiberaceae, PMP-669. An essential oil percentage and the total content of phenolic and flavonoid compounds of green cardamom will be assessed using high-performance liquid chromatography (HPLC) and gas chromatography mass spectrometry (GC-MS) in the institute of medicinal plants, Shahid Beheshti University of Medical Sciences, Tehran, Iran. Some polyphenolic compounds of green cardamom, whose effects have been mentioned in other articles (caffeic acid, gallic acid, quercetin and luteolin), will also be determined by using HPLC.

Supplements are to be distributed among patients once a month and their potential complications and consumption processes registered (number of consumed capsules and the returned packages). In addition, the consumption process is to be checked once a week, by telephone.

### Lifestyle changes

A weight-loss diet (5% weight loss during the study), recommendations for increasing physical activity (aerobic with moderate intensity, at least three times per week at 30–45 min), and lifestyle changes will be presented to all patients enrolled for the study by an experienced dietician placed in the polyclinic of the NIOC Central Hospital in Tehran.

### Measurements and assessments

The grade of fatty liver is to be determined in a fasting state by medical ultrasound (also known as diagnostic sonography or ultrasonography). This is a diagnostic imaging technique which applies ultrasound in order to examine the images of internal bodily organs and structures such as the liver. Its aim is often to find the source of a disease or to exclude any pathology. One radiologist will do the ultrasound diagnostic imaging for all patients to reduce human error differences.

Serum lipid profile (TC, HDL, LDL, TG) and liver enzymes (alanine transaminase (ALT), aspartate transaminase (AST)) are to be measured by using the specific kits and Hitachi analyzer (or BT-3500) device. Serum lipid profile has now become almost a routine test. These will be done in a 12-h fasting state.

Blood glucose profiles, including FBS, FBI, HOMA-IR, and QUICKI are measured by a glucose specific kit (glucose oxidase method), electrochemiluminescence ((ECL) by the cobas e *411*® analyzer device) and the following formulae, respectively:

$$ \mathrm{QUICKI}=1/\left( \log\ \left(\mathrm{fasting}\ \mathrm{insulin}\kern0.5em \mu \mathrm{U}/\mathrm{ml}\right)+ \log\ \left(\mathrm{fasting}\ \mathrm{glucose}\ \mathrm{mg}/\mathrm{dl}\right)\right) $$

$$ \mathrm{HOMA}\hbox{-} \mathrm{I}\mathrm{R}=\left(\mathrm{FBI}\ \left(\mathrm{mU}/\mathrm{l}\right)\times \mathrm{FBS}\ \left(\mathrm{mmol}/\mathrm{l}\right)\right)/22.5 $$

Glucose oxidase is widely used for the determination of free glucose level in body diagnostic fluids.

Serum inflammatory factors (IL-6, TNF-α, hs-CRP), irisin, Sirt1 and PON1 are measured by using the enzyme-linked immunosorbent assay (ELISA) method (sandwich ELISA format) and specific kits.

The ELISA is a test that uses antibodies and color change to identify a substance. The ELISA is often used as a diagnostic tool in medicine [54]. The ELISA test will be done by Elisa washer (Combiwash Human®) and bioElisa reader devices (biokit® ELx800) in this study.

Food intake and physical activity are to be assessed by using a 24-h food recall (at the beginning, after 1.5 months, and at the end) questionnaire and short-form IPAQ (at the beginning and the end), respectively.

The patients will be asked to remember all consumed food and drink during the past 24 h when completing the 24-h food recall questionnaire. This questionnaire has previously been validated in Iran [55]. The intake values will be turned to g/day, based on household food scales [56]. The dietary intakes are to be calculated by using the DFP (Dorosty Food Processor) software that contains Iranian food composition tables [55]. The intake of macronutrients and micronutrients including dietary antioxidants will thus be determined.

The purpose of the IPAQ is to provide a set of well-developed instruments that can be used internationally to obtain comparable estimates of physical activity. The short version of the questionnaire is suitable for use in national and regional surveillance systems and provides information required in research work or for evaluation purposes. Three levels (categories) of physical activity are proposed: low, moderate, and high [57]. This questionnaire has been validated in previous studies [58–60] including in Iran [61, 62].

The effect of dietary intakes, including dietary antioxidants and physical activity, will be controlled by prescribing the same diet and physical activity for all patients in both groups.

Systolic and diastolic blood pressure values will be measured with a mercury manometer at the beginning and the end of the study. Values are to be recorded in millimeters of mercury (mmHg).

Weight, height and waist circumference are determined by using a digital scale, stadiometer and nonelastic tape, respectively. They are measured thus: weight without shoes, with minimal clothing, and with a 100-g accuracy; height without shoes, standing, heels against the wall, flat and forward head, and with 0.5-cm accuracy; and waist circumference with minimal clothing, midway between the last rib and the iliac crest.

Collection of blood, specimen storage, and laboratory tests are to be conducted at the NIOC Central Hospital in Tehran.

**Fig. 1:**
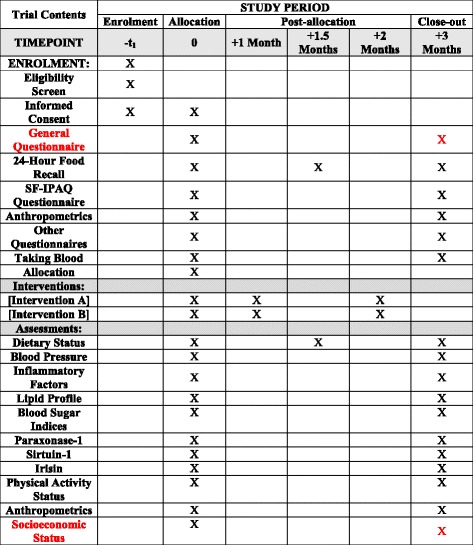
Contents include enrollment, interventions, and assessments

Figure 1 presents the overall contents of enrollment, interventions, and assessments. Moreover, the Standard Protocol Items: Recommendations for Interventional Trials (SPIRIT) Checklist is provided as an Additional file 1.

The trial conduct is to be frequently audited by an assistant through an independent process.

### Data analysis

Data entry, coding, security, and storage are also to be considered. The normality of continuous variables will be determined using a Kolmogorov-Smirnov test. Continuous and categorical baseline characteristics of the groups will be assessed using a *t* test and a chi-square test, respectively. Multivariate, two-factor, repeated measures analysis of variance will be used to assess time effects and to analyze time × treatment interaction effects on all dependent variables. All statistical measurements will be reported with 95% confidence interval (CI). A *P* value < 0.05 will be considered as statistically significant. The data will be analyzed by using SPSS software.

## Data accessibility

The principal investigator will have access to the final trial dataset, and such access for other investigators is limited. The trial results are presented only through the publication.

## Discussion

This trial will first assess the effects of green cardamom (*Elettaria cardamomum*) on blood glucose indices, lipids, inflammatory factors, paraxonase-1, sirtuin-1 and irisin in overweight or obese patients with nonalcoholic fatty liver disease (NAFLD). The different clinical usages and the lack of awareness concerning the advantages and disadvantages of green cardamom in patients with NAFLD makes this study very pertinent. Although there is a hypothetical reason for using green cardamom in the treatment of some disorders, its therapeutic use in humans necessitates the study of its potential in different diseases, particularly NAFLD. Attention should be paid to the increasing rates of obesity (and, consequently, NAFLD). Due to remarkable changes of some blood markers in this population, and the lack of human studies on the effects of green cardamom, this trial selected these patients as the most suitable cases for intervention.

The strengths of the study are its randomized double-blinded design, protocol publication, the measurement of dietary outcomes and physical activity status, and the recording of patient-reported probable complications.

The limitations of the study are self-reporting of diet and physical activity, slow patient recruitment because of the eligibility criteria, a single center and lack of cooperation by some patients at the end, which may lead to these patients needing to be being replaced.

## Trial status

Recruitment of the participants was ongoing at the time of manuscript submission.

## Additional file

Additional file 1: SPIRIT Checklist. This checklist applies to protocols for all clinical trials and focuses on content rather than format. The checklist recommends a full description of what is planned; it does not prescribe how to design or conduct a trial. The SPIRIT recommendations aim to facilitate the drafting of high-quality protocols and enhance the transparency and completeness of trial protocols for the benefit of investigators, trial participants, patients, sponsors, funders, research ethics committees or institutional review boards, peer reviewers, journals, trial registries, policymakers, regulators, and other key stakeholders. Click here for file: www.spirit-statement.org/wp-content/uploads/2013/08/SPIRIT-Checklist-download-8Jan13.doc. (DOC 138 kb)

## Abbreviations

ALT: Alanine transaminase
ANCOVA: Analysis of covariance
AST: Aspartate transaminase
BMI: Body Mass Index
CNS: Central nervous system
COX-2: Cyclooxygenase-2
DFP: Dorosty Food Processor
ECL: Electrochemiluminescence
ELISA: Enzyme-linked immunosorbent assay
FBI: Fasting blood insulin
FBS: Fasting blood sugar
FGF-21: Fibroblast growth factor-21
GC-MS: Gas chromatography mass spectrometry
HDL-c: High-density lipoprotein-cholesterol
HOMA-IR: Homeostasis model assessment-insulin resistance
HPLC: High-performance liquid chromatography
hs-CRP: High-sensitivity C-reactive protein
IL-1β: Interleukin-1 beta
IL-6: Interleukin-6
iNOS: Inducible nitric oxide synthase
IPAQ: International Physical Activity Questionnaire
LDL-c: Low-density lipoprotein-cholesterol
NAFLD: Nonalcoholic fatty liver disease
NASH: Nonalcoholic steatohepatitis
NF-κB: Nuclear factor kappa-light-chain-enhancer of activated B-cells
NIOC: National Iranian Oil Company
PGC-1α: PPAR-γ co-activator-1 alpha
PON-1: Paraxonase-1
PPAR: Peroxisome proliferation-activated receptor
QUICKI: Quantitative Insulin Sensitivity Check Index
ROS: Reactive oxygen species
Sirt-1: Sirtuin-1
SOD: Superoxide dismutase
TC: Total cholesterol
TG: Triglyceride
TMRC: Traditional Medicine Research Center
TNF-α: Tumor necrosis factor-alpha
TPN: Total parenteral nutrition
